# Comparison of the efficacy of nafcillin and glycopeptides as definitive therapy for patients with methicillin-susceptible *Staphylococcus aureus* bacteremia: a retrospective cohort study

**DOI:** 10.1186/s12879-018-2978-z

**Published:** 2018-01-30

**Authors:** Dong Hyun Oh, Jung Ju Kim, Jinnam Kim, Hye Seong, Se Ju Lee, Yong Chan Kim, Eun Jin Kim, In Young Jung, Woo Yong Jeong, Su Jin Jeong, Nam Su Ku, Sang Hoon Han, Jun Yong Choi, Young Goo Song, June Myung Kim

**Affiliations:** 1 Department of Internal Medicine, Seoul Medical Center, 156 Sinnae-ro, Jungnang-gu, 02053 Seoul, South Korea; 2 Department of Internal Medicine, Yonsei University College of Medicine, 50-1 Yonsei-ro, Seodaemun-gu, 03722 Seoul, Republic of Korea; 3 AIDS Research Institute, Yonsei University College of Medicine, 50-1 Yonsei-ro, Seodaemun-gu, 03722 Seoul, South Korea

**Keywords:** Methicillin-susceptible *Staphylococcus aureus*, Nafcillin, Antistaphylococcal penicillin, Glycopeptides

## Abstract

**Background:**

Studies have shown that the prognosis of the treatment of methicillin-susceptible *S. aureus* (MSSA) with glycopeptides is inferior compared to treatment with β-lactam. However, there are only few studies comparing treatment with antistaphylococcal penicillin alone to glycopeptide treatment. The aim of this study was to compare the efficacy of nafcillin, an antistaphylococcal penicillin, with that of glycopeptides as a definitive therapy for MSSA bacteremia.

**Methods:**

Patients with MSSA bacteremia recruited from a tertiary referral hospital were enrolled in this retrospective cohort study. Demographic characteristics, laboratory data, and clinical outcome of the treatment were compared between a group receiving nafcillin and a group receiving glycopeptides.

**Results:**

A total of 188 patients with MSSA bacteremia were included in this study. The glycopeptide group had a higher rate of malignancy (28.6 vs. 60.8%, *p* < 0.001) and proportion of healthcare-associated infections (47.3 vs. 72.2%, *p* < 0.001) compared to the nafcillin group. The ratio of skin and soft tissue infections (30.0 vs. 16.7%, *p* = 0.037) and bone and joint infections (17.8 vs. 6.3%, *p* = 0.022), as well as levels of C-reactive protein (139.60 vs. 107.61 mg/dL, *p* = 0.022) were higher in the nafcillin group. All-cause 28-day mortality was significantly high in the glycopeptide group (7.7 vs. 20.6%, *p* = 0.013).

**Conclusion:**

In patients with MSSA bacteremia, all-cause 28-day mortality rate was higher in a group treated with glycopeptides than in a group treated with nafcillin. Therefore, the use of nafcillin should be considered as a definitive therapy for MSSA bacteremia.

**Electronic supplementary material:**

The online version of this article (10.1186/s12879-018-2978-z) contains supplementary material, which is available to authorized users.

## Background

*Staphylococcus aureus* (*S. aureus*) is one of the most common causes of community-acquired and healthcare-associated bacteremia [1]. The incidence of infection induced by methicillin-resistant *S. aureus* (MRSA) has been increasing [2, 3]. In Korea, a recent nationwide survey reported a methicillin-resistance rate of *S. aureus* of 66% in the general population [4]. Therefore, many clinicians therefore use glycopeptides such as vancomycin as their first choice of antibiotics for empirical treatment of *S. aureus* bacteremia to cover both MRSA and methicillin-susceptible *S. aureus* (MSSA) before confirming antibiotic susceptibility. However, some physicians continue to use the glycopeptide without changing to β-lactam antibiotics after confirmation of MSSA bacteremia.

Many studies comparing the efficacy of β-lactam antibiotics, including antistaphylococcal penicillin and glycopeptides, as empirical therapy for MSSA bacteremia have been conducted, and β-lactam agents have been found to be superior in the treatment of MSSA bacteremia compared with glycopeptides in these studies [5–8]. Wong et al. reported that empiric β-lactams was associated with earlier clearance of MSSA bacteremia compared to vancomycin (70.7 vs. 97.1 h, *p* = 0.007) [5]. Additionally, Schweizer et al. reported that β-lactams showed protective effectiveness against mortality compared to vancomycin in patients with MSSA bacteremia (adjusted hazard ratio, 0.21; 95% CI, 0.09–0.47) [9]. However, there were few studies comparing glycopeptides and antistaphylococcal penicillin such as nafcillin alone as a treatment for MSSA bacteremia. This study aimed to compare the efficacy of nafcillin, an antistaphylococcal antibiotic, with that of glycopeptides including vancomycin and teicoplanin, as definitive therapy for patients with MSSA bacteremia.

## Methods

### Study population

We consecutively included patients with confirmed *S. aureus* bacteremia recruited from a single tertiary referral hospital from April 2012 to June 2016 in this retrospective cohort study. The referral hospital is a 2400-bed institution affiliated with Yonsei University College of Medicine in Seoul, South Korea. All participants were aged 18 years or older. Patients receiving a definitive therapy with nafcillin, vancomycin, or teicoplanin were included in this study. Patients who had MRSA infections or received treatment with other single antibiotics such as first generation cephalosporin, quinolone, or β-lactam/β-lactamase inhibitor combinations were excluded. Patients with bacteremia caused by other pathogens and those who died before the causative organism could be confirmed were also excluded. Participants were divided into the “nafcillin group” and the “glycopeptide group”. The study was approved by the institutional review board of the Yonsei University Health System Clinical Trial Center (#4–2017-0070).

### Data collection

Baseline characteristics such as age, sex, and preexisting comorbidities including cardiovascular disease, cerebral vascular accidents, dementia, lung disease, autoimmune disease, peptic ulcer disease, chronic kidney disease, diabetes, liver disease, and malignancy were recorded. Data on the source of the bacteremia and on whether the patient’s infection was community-acquired or healthcare-associated were collected. According to the source of bacteremia, we subdivided the patient sample into groups with catheter-related infection, pneumonia, urinary tract infection, skin and soft tissue infection, bone and joint infection, intra-abdominal infection, and primary bacteremia.

Laboratory tests were undertaken within the first 24 h after a culture was drawn. Tests included white blood cell (WBC) count, platelet counts, creatinine levels, estimated glomerular filtration rate, total bilirubin levels, prothrombin time (international normalized ratio), and C-reactive protein (CRP) levels. As the index of disease severity, the Pitt bacteremia score was calculated [10]. To assess the clinical outcome, we analyzed the patient data with respect to hospitalization duration, whether or not participants received intensive care unit (ICU) care, duration of stay at the ICU, persistent bacteremia, and all-cause 28-day mortality. The results of an antimicrobial susceptibility test, performed in accordance with the Clinical & Laboratory Standards Institute guidelines, were obtained from the patients’ medical records [11].

### Definition

If bacteremia was identified within 48 h of admission and the participant had no history of admission to healthcare institutions within 3 months prior to admission, their infection was assumed to be community-acquired. If bacteremia was identified after 48 h or if the participant had an admission history within 3 months prior to admission, their infection was assumed to be healthcare-associated. Definitive therapy was defined as initiated or continued antibiotic treatment after identification of the pathogen in blood culture and in an antibiotic susceptibility test [12]. The source of infection, such as catheter-related infection, pneumonia, urinary tract infection, skin and soft tissue infection, bone and joint infection, and intra-abdominal infection was defined based on the criteria laid out by the Centers for Disease Control and Prevention, USA [13]. WBC counts equal to or above 10,000/mm^3^ are considered leukocytosis. Leukopenia is defined as WBC counts under 4000/mm^3^. Thrombocytopenia is defined as platelet counts less than 150 × 10^3^/mm^3^. Persistent bacteremia is defined as the isolation of *S. aureus* in blood cultures obtained from peripheral veins for more than 7 consecutive days despite adequate antibiotic administration for more than 5 days. Mortality involving at least one of the following are defined as bacteremia-related mortality: (i) blood cultures were positive at the time of death; (ii) death occurred before the resolution of bacteremia; (iii) death occurred during hospitalization without other specific cause except bacteremia [14].

### Statistical analysis

Continuous variables were expressed as mean ± standard deviation or median (interquartile range) and compared using the Student’s *t*-test if the variables followed a normal distribution. Continuous variables with skewed distribution were compared using the Mann-Whitney *U*-test. The chi-squared test was used when all the categorical variables included in the analysis were 5 or more. If any of the categorical variables included in the analysis has a value of less than 5, it was analyzed using Fisher’s exact test. To compare survival rates between the nafcillin group and the glycopeptide group, a Kaplan-Meier survival curve was used. The Cox regression model was used to analyze prognostic factors for mortality. All statistical analyses were performed using the Statistical Package for the Social Sciences 18.0 software (SPSS Inc., Chicago, IL, USA). *P* values under 0.05 were considered statistically significant.

## Results

**Fig. 1:**
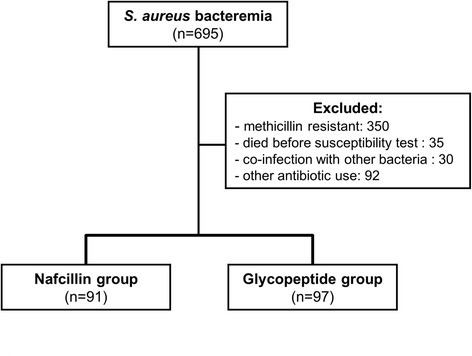
Study flow diagram

A total of 695 patients with *S. aureus* bacteremia were identified for screening. Three hundred and fifty patients with MRSA infection were excluded, and 35 patients who died before antimicrobial susceptibility was confirmed were also excluded. Additionally, 30 patients with coinfections with other bacteria and 92 patients treated with other antibiotics were excluded. Among the remaining 280 patients, 188 patients who underwent treatment with nafcillin, vancomycin, or teicoplanin were finally enrolled in this study. Ninety-one patients received nafcillin, and 97 patients received glycopeptides (vancomycin or teicoplanin) as definitive therapy (Fig. 1).

The mean age of the study participants was 62.37 ± 15.14 years, and 69% (*n* = 129) of all patients were male. With regard to underlying diseases, the patients in the glycopeptide group had a significantly higher rate of malignancy than the patients in the nafcillin group (28.6 vs. 60.8%, *p* < 0.001). There was no significant difference with regard to other comorbidities. Additionally, the patients in the glycopeptide group had a higher proportion of healthcare-associated infections (47.3 vs. 72.2%, *p* < 0.001). The most common infection source was skin and soft tissue infection (23.1%), followed by catheter-related infection and bone and joint infection (11.8%, respectively). Skin and soft tissue infections (30.0 vs. 16.7%, *p* = 0.037) and bone and joint infections were common in the nafcillin group. Laboratory tests showed that the median CRP level was significantly higher in the nafcillin group than in the glycopeptide group (139.60 vs. 107.61 mg/dL, *p* = 0.022). There was no significant difference in other laboratory tests (Table 1).

**Table 1:** Comparison of baseline characteristics and laboratory test results between the patient groups treated with either nafcillin or glycopeptides

Characteristics	All patients (*n* = 188)	Nafcillin group (*n* = 91)	Glycopeptide group (*n* = 97)	*P* value
Age, years, mean ± SD	62.37 ± 15.14	64.38 ± 14.53	60.46 ± 15.54	0.076 ^a^
Male, (%)	129 (69.0)	61 (67.0)	68 (70.8)	0.636 ^b^
Underlying comorbidities, yes (%)				
Cardiovascular disease	55 (29.3)	29 (31.9)	26 (26.8)	0.522 ^b^
Cerebral vascular accident	19 (10.1)	12 (13.2)	7 (7.2)	0.227 ^b^
Dementia	5 (2.7)	1 (1.1)	4 (4.1)	0.370 ^c^
Lung disease	14 (7.4)	8 (8.8)	6 (6.2)	0.584 ^b^
Autoimmune disease	10 (5.3)	4 (4.4)	6 (6.2)	0.749 ^c^
Peptic ulcer disease	10 (5.3)	6 (6.6)	4 (4.1)	0.527 ^c^
Chronic kidney disease	39 (20.7)	23 (25.3)	16 (16.5)	0.153 ^b^
Diabetes	62 (33.0)	35 (38.5)	27 (27.8)	0.162 ^b^
Liver disease	23 (12.2)	8 (8.8)	15 (15.5)	0.187 ^b^
Malignancy	85 (45.2)	26 (28.6)	59 (60.8)	<0.001 ^b^
CA vs. HCA, HCA (%)	113 (60.1)	43 (47.3)	70 (72.2)	<0.001 ^b^
Pit bacteremia score, mean ± SD	1.56 ± 2.55	1.42 ± 2.39	1.69 ± 2.71	0.465 ^a^
Infection focus, yes (%)				
Catheter-related infection	22 (11.8)	12 (13.3)	10 (10.4)	0.651 ^b^
Pneumonia	8 (4.3)	1 (1.1)	7 (7.3)	0.066 ^c^
Urinary tract infection	7 (3.8)	9 (6.7)	1 (1.0)	0.058 ^c^
Skin and Soft tissue infection	43 (23.1)	27 (30.0)	16 (16.7)	0.037 ^b^
Bone and Joint infection	22 (11.8)	16 (17.8)	6 (6.3)	0.022 ^b^
Intra-abdominal infection	13 (7.0)	3 (3.3)	10 (10.4)	0.083 ^c^
Primary bacteremia	74 (39.8)	27 (30.0)	47 (49.0)	0.011 ^b^
Laboratory tests				
WBC, /mm^3^, median (IQR)	10,150 (7,635–14,770)	10,450 (8,120–15,280)	9,860 (5,485–14,615)	0.064 ^d^
Leukocytosis or Leukopenia, yes (%)	116 (61.7)	50 (54.9)	66 (68.0)	0.073 ^b^
Platelet counts, ×10^3^/mm^3^, median (IQR)	169 (95–250)	182 (115–257)	164 (83–250)	0.190 ^d^
Thrombocytopenia, yes (%)	82 (43.6)	36 (39.6)	46 (47.4)	0.305 ^b^
eGFR, mL/min/mm^3^, mean ± SD	62.88 ± 28.60	62.38 ± 28.27	63.35 ± 29.06	0.818 ^a^
Total bilirubin, mg/dL, median (IQR)	0.8 (0.5–1.4)	0.8 (0.5–1.3)	0.8 (0.5–1.4)	0.452 ^d^
Prothrombin time (INR), median (IQR)	1.12 (1.01–1.29)	1.13 (1.02–1.28)	1.11 (1.01–1.32)	0.895 ^d^
CRP, mg/dL, median (IQR)	121.01 (56.34–200.85)	139.60 (79.68–219.00)	107.61 (42.19–192.90)	0.022 ^d^

Abbreviations: *SD* standard deviation, *CA* community-acquired, *HCA* healthcare-associated, *WBC* white blood cell, *IQR* interquartile range, *eGFR* estimated glomerular filtration rate, *INR* international normalized ratio, *CRP* C-reactive protein

^a^Student’s *t*-test

^b^Pearson’s χ-test

^c^Fisher’s exact test

^d^Mann-Whitney *U*-test, median (interquartile range)

With regard to the antimicrobial susceptibility of identified MSSA, we found a relatively high resistance rate to penicillin G (85.1%), erythromycin (17.1%), and clindamycin (15.4%). Resistance to other antibiotics was relatively low. There was no significant difference in resistance rates between the two patient groups except for clindamycin (8.8 vs. 21.6%, *p* = 0.016) (Additional file 1).

**Fig. 2:**
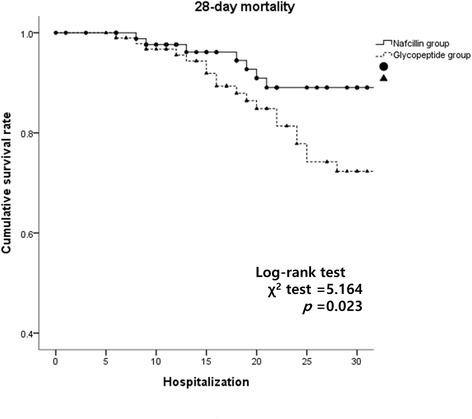
Comparison of all cause 28-day mortality between nafcillin group and glycopeptide group by Kaplan-Meier method and Log-rank test

The median hospitalization period and the mean duration of stay at the ICU of all participants were 22.0 (14.0–39.5) days and 5.03 ± 14.10 days, respectively. The rate of persistent bacteremia was 9.6%. There was no difference between the two groups in terms of hospitalization period (22.0 vs. 22.0 days, *p* = 0.764). There were no significant differences in the duration of ICU stay (3.71 vs. 6.26 days, *p* = 0.217) and in the ratio of persistent bacteremia (12.2 vs. 7.2%, *p* = 0.323) between the two patient groups. (Table 2, Fig. 2). There was a significant difference between the two patient groups in all-cause 28-day mortality (7.7 vs. 20.6%, *p* = 0.013) and bacteremia-related 28-day mortality (4.4 vs. 14.4%, *p* = 0.025). Additionally, the Cox regression analysis showed that the use of glycopeptides was the significant prognostic factor for mortality when adjusting for malignancy, healthcare-associated infection, and CRP (Hazard ratio, 2.615; 95% CI, 1.105–6.186, *p* = 0.029) (Additional file 2).

**Table 2:** Clinical outcome of the different treatments

Characteristics	All patients (*n* = 188)	Nafcillin group (*n* = 91)	Glycopeptide group (*n* = 97)	*P* value
Hospitalization period, days, median (IQR)	22.0 (14.0–39.5)	22.0 (12.0–41.0)	22.0 (15.0–36.0)	0.764^c^
ICU stay, days, mean ± SD	5.03 ± 14.10	3.71 ± 12.47	6.26 ± 15.44	0.217^a^
Persistent bacteremia, (%)	18 (9.6)	11 (12.2)	7 (7.2)	0.323^b^
All-cause 28-day mortality, yes (%)	27 (14.4)	7 (7.7)	20 (20.6)	0.013^b^

Abbreviations: *IQR* interquartile range, *ICU* intensive care unit, *SD* standard deviation

^a^Student’s *t*-test

^b^Pearson’s χ-test

^c^Mann-Whitney *U*-test, median (interquartile range)

## Discussion

*S. aureus* bacteremia is a very severe clinical condition that clinicians often experience. Since the infection rate caused by MRSA is increasing, many clinicians use the glycopeptide empirically. However, despite of confirming MSSA bacteremia via bacterial culture, it is not uncommon to maintain glycopeptide as definitive therapy for the reason that severe comorbidities and disease severity. There have been many studies related to the choice of a definitive therapeutic agent for MSSA bacteremia, but no study had compared prognosis with glycopeptide with nafcillin alone, an antistaphylococcal agent. In our current study, we found that the 28-day mortality rate was significantly lower in a group of patients with MSSA bacteremia receiving definitive therapy with nafcillin compared to a group receiving treatment with. This finding suggests that nafcillin should be considered as the first choice for definitive antimicrobial therapy in MSSA bacteremia.

Our finding of higher mortality in the vancomycin group is in accordance with findings from several recent studies. Mcdanel et al. reported a higher mortality rate in patients who received vancomycin monotherapy compared with patients who received β-lactam monotherapy, as definitive therapy for MSSA bacteremia, after adjustment for confounding factors such as severity of illness, comorbidities, age, and others [7]. Kim et al. reported higher mortality in patients who were treated with vancomycin for MSSA bacteremia compared with patients receiving β-lactam agents [6].

Some explanations for the relative inferiority of vancomycin in the treatment of MSSA have been proposed [15]. Generally, β-lactam agents are classified as bactericidal to MSSA, whereas the bactericidal effect of vancomycin is lower than that of β-lactam agents [15, 16]. Furthermore, vancomycin has a relatively narrow therapeutic range as compared to β-lactam agents, pharmacologically [15]. Additionally, because teicoplanin is a bacteriostatic agent with similar efficacy as vancomycin, teicoplanin also poses problems that is smilar with vancomycin in the treatment of MSSA [17].

In this study, the prevalence of malignancy and the proportion of healthcare-associated infections were higher in the glycopeptide group than in the nafcillin group. In addition, skin and soft tissue infection as well as bone and joint infection were more common in the nafcillin group than in the glycopeptide group. However, the use of glycopeptides was found to be the significant prognostic factor for mortality when adjusting for these variables. Thus, despite several confounding variables, the choice of antibiotics, in this case nafcillin versus glycopeptides, in the treatment of MSSA bacteremia is an important factor affecting patient mortality.

On the other hand, we found no significant difference in persistent bacteremia in this study. Kim et al. reported that there was no statistical difference in eradication of infection foci between a β-lactam treatment group and a vancomycin treatment group in patients with MSSA bacteremia [6]. However, Wong *el al.* reported a significant difference in the frequency of prolonged bacteremia between a β-lactam treatment group and a vancomycin treatment group [5]. Furthermore, Park et al. reported significant differences in bacteremia duration between a β-lactam treatment group, a vancomycin treatment group, and a combination treatment group [18].

This study has several limitations. First, this is a retrospective cohort study and it is thus subject to selection bias. Second, all data were collected from a single center, so that generalization of these results to other institutions might be questionable. Additional prospective studies with more selective and larger populations involving multiple centers are necessary.

## Conclusion

In conclusion, we find that all-cause 28-day mortality in MSSA bacteremia is higher in patients receiving glycopeptides as definitive therapy compared to patients receiving nafcillin. Therefore, the use of nafcillin should be a first choice for definitive antimicrobial treatment in MSSA bacteremia.

## Additional files

Additional file 1: **Supplement 1.** Antibiotic resistance rate of identified *Staphylococcus aureus*. (DOCX 21 kb)

Additional file 2: Multivariate analysis of prognostic factors for predicting mortality in patients with MSSA bacteremia, with adjustments for prevalence of malignancy, healthcare-associated infections, and CRP. (DOCX 13 kb)
