# Supplementary material for: Comparison of the efficacy of nafcillin and glycopeptides as definitive therapy for patients with methicillin-susceptible Staphylococcus aureus bacteremia: a retrospective cohort study
Source: BMC Infect Dis. 2018 Jan 30;18:60. doi: 10.1186/s12879-018-2978-z (PMC5789670; doi:10.1186/s12879-018-2978-z)
Supplement: Supplementary file 1 — Supplement 1. Antibiotic resistance rate of identified Staphylococcus aureus. (DOCX 21 kb) [file 12879_2018_2978_MOESM1_ESM.docx]

**Table 1.** Comparison of baseline characteristics and laboratory test results between the patient groups treated with either nafcillin or glycopeptides.

| **Characteristics** | | **All patients**  **(n = 188)** | **Nafcillin group**  **(n = 91)** | | **Glycopeptide group**  **(n = 97)** | | ***P* value** | |  |
| --- | --- | --- | --- | --- | --- | --- | --- | --- | --- |
| Age, years, mean ± SD | | 62.37 ± 15.14 | 64.38 ± 14.53 | | 60.46 ± 15.54 | | 0.076 ^a^ | |  |
| Male, (%) | | 129 (69.0) | 61 (67.0) | | 68 (70.8) | | 0.636 ^b^ | |  |
| Underlying comorbidities, yes (%) | |  |  | |  | |  | |  |
| Cardiovascular disease | | 55 (29.3) | 29 (31.9) | | 26 (26.8) | | 0.522 ^b^ | |  |
| Cerebral vascular accident | | 19 (10.1) | 12 (13.2) | | 7 (7.2) | | 0.227 ^b^ | |  |
| Dementia | | 5 (2.7) | 1 (1.1) | | 4 (4.1) | | 0.370 ^c^ | |  |
| Lung disease | | 14 (7.4) | 8 (8.8) | | 6 (6.2) | | 0.584 ^b^ | |  |
| Autoimmune disease | | 10 (5.3) | 4 (4.4) | | 6 (6.2) | | 0.749 ^c^ | |  |
| Peptic ulcer disease | | 10 (5.3) | 6 (6.6) | | 4 (4.1) | | 0.527 ^c^ | |  |
| Chronic kidney disease | | 39 (20.7) | 23 (25.3) | | 16 (16.5) | | 0.153 ^b^ | |  |
| Diabetes | | 62 (33.0) | 35 (38.5) | | 27 (27.8) | | 0.162 ^b^ | |  |
| Liver disease | | 23 (12.2) | 8 (8.8) | | 15 (15.5) | | 0.187 ^b^ | |  |
| Malignancy | | 85 (45.2) | 26 (28.6) | | 59 (60.8) | | <0.001 ^b^ | |  |
| CA vs. HCA, HCA (%) | | 113 (60.1) | 43 (47.3) | | 70 (72.2) | | <0.001 ^b^ | |  |
| Pit bacteremia score, mean ± SD | | 1.56 ± 2.55 | 1.42 ± 2.39 | | 1.69 ± 2.71 | | 0.465 ^a^ | |  |
| Infection focus, yes (%) | |  |  | |  | |  | |  |
| Catheter-related infection | | 22 (11.8) | 12 (13.3) | | 10 (10.4) | | 0.651 ^b^ | |  |
| Pneumonia | | 8 (4.3) | 1 (1.1) | | 7 (7.3) | | 0.066 ^c^ | |  |
| Urinary tract infection | | 7 (3.8) | 9 (6.7) | | 1 (1.0) | | 0.058 ^c^ | |  |
| Skin and Soft tissue infection | | 43 (23.1) | 27 (30.0) | | 16 (16.7) | | 0.037 ^b^ | |  |
| Bone and Joint infection | | 22 (11.8) | 16 (17.8) | | 6 (6.3) | | 0.022 ^b^ | |  |
| Intra-abdominal infection | | 13 (7.0) | 3 (3.3) | | 10 (10.4) | | 0.083 ^c^ | |  |
| Primary bacteremia | | 74 (39.8) | 27 (30.0) | | 47 (49.0) | | 0.011 ^b^ | |  |
| Laboratory tests |  | | |  | |  | |  | |
| WBC, /mm^3^, median (IQR) | 10,150 (7,635–14,770) | | | 10,450 (8,120–15,280) | | 9,860 (5,485–14,615) | | 0.064 ^d^ | |
| Leukocytosis or Leukopenia, yes (%) | 116 (61.7) | | | 50 (54.9) | | 66 (68.0) | | 0.073 ^b^ | |
| Platelet counts, ×10^3^/mm^3^, median (IQR) | 169 (95–250) | | | 182 (115–257) | | 164 (83–250) | | 0.190 ^d^ | |
| Thrombocytopenia, yes (%) | 82 (43.6) | | | 36 (39.6) | | 46 (47.4) | | 0.305 ^b^ | |
| eGFR, mL/min/mm^3^, mean ± SD | 62.88 ± 28.60 | | | 62.38 ± 28.27 | | 63.35 ± 29.06 | | 0.818 ^a^ | |
| Total bilirubin, mg/dL, median (IQR) | 0.8 (0.5–1.4) | | | 0.8 (0.5–1.3) | | 0.8 (0.5–1.4) | | 0.452 ^d^ | |
| Prothrombin time (INR), median (IQR) | 1.12 (1.01–1.29) | | | 1.13 (1.02–1.28) | | 1.11 (1.01–1.32) | | 0.895 ^d^ | |
| CRP, mg/dL, median (IQR) | 121.01 (56.34–200.85) | | | 139.60 (79.68–219.00) | | 107.61 (42.19–192.90) | | 0.022 ^d^ | |

Abbreviations: SD: standard deviation; CA: community-acquired; HCA: healthcare-associated; WBC: white blood cell; IQR: interquartile range; eGFR: estimated glomerular filtration rate; INR: international normalized ratio; CRP: C-reactive protein.

^a^ Student’s *t*-test

^b^ Pearson’s χ-test

^c^ Fisher’s exact test

^d^ Mann-Whitney *U*-test, median (interquartile range)
