# Supplementary material for: Comparison of the efficacy of nafcillin and glycopeptides as definitive therapy for patients with methicillin-susceptible Staphylococcus aureus bacteremia: a retrospective cohort study
Source: BMC Infect Dis. 2018 Jan 30;18:60. doi: 10.1186/s12879-018-2978-z (PMC5789670; doi:10.1186/s12879-018-2978-z)
Supplement: Supplementary file 2 — Multivariate analysis of prognostic factors for predicting mortality in patients with MSSA bacteremia, with adjustments for prevalence of malignancy, healthcare-associated infections, and CRP. (DOCX 13 kb) [file 12879_2018_2978_MOESM2_ESM.docx]

**Table 2.** Clinical outcome of the different treatments.

| **Characteristics** | **All patients**  **(n = 188)** | **Nafcillin group**  **(n = 91)** | **Glycopeptide group**  **(n = 97)** | ***P* value** |
| --- | --- | --- | --- | --- |
| Hospitalization period, days, median (IQR) | 22.0 (14.0–39.5) | 22.0 (12.0–41.0) | 22.0 (15.0–36.0) | 0.764 ^c^ |
| ICU stay, days, mean ± SD | 5.03 ± 14.10 | 3.71 ± 12.47 | 6.26 ± 15.44 | 0.217 ^a^ |
| Persistent bacteremia, (%) | 18 (9.6) | 11 (12.2) | 7 (7.2) | 0.323 ^b^ |
| All-cause 28-day mortality, yes (%) | 27 (14.4) | 7 (7.7) | 20 (20.6) | 0.013 ^b^ |

Abbreviations: IQR: interquartile range; ICU: intensive care unit; SD: standard deviation.

^a^ Student’s *t*-test

^b^ Pearson’s χ-test

^c^ Mann-Whitney *U*-test, median (interquartile range)
